# Effects of Dietary Plant Sterols and Stanol Esters with Low- and High-Fat Diets in Chronic and Acute Models for Experimental Colitis

**DOI:** 10.3390/nu7105412

**Published:** 2015-10-15

**Authors:** Anje A. te Velde, Florence Brüll, Sigrid E. M. Heinsbroek, Sybren L. Meijer, Dieter Lütjohann, Anita Vreugdenhil, Jogchum Plat

**Affiliations:** 1 Tytgat Institute for Liver and Intestinal Research, Amsterdam Medical Centre, Amsterdam 1105 BK, The Netherlands; s.e.heinsbroek@amc.uva.nl; 2 Department of Human Biology or Pediatrics, School for Nutrition, Toxicology and Metabolism, Maastricht University, Maastricht 6229 ER, The Netherlands; florence.brull@maastrichtuniversity.nl (F.B.); a.vreugdenhil@mumc.nl (A.V.); j.plat@maastrichtuniversity.nl (J.P.); 3 Department of Pathology, Amsterdam Medical Centre, Amsterdam 1105 AZ, The Netherlands; s.l.meijer@amc.uva.nl; 4 Institute of Clinical Chemistry and Clinical Pharmacology, University of Bonn, Bonn D-53127, Germany; Dieter.Luetjohann@ukb.uni-bonn.de

**Keywords:** experimental colitis, nutrition, plant sterols, plant stanols, inflammation, T cells

## Abstract

In this study, we evaluated the effects of dietary plant sterols and stanols as their fatty acid esters on the development of experimental colitis. The effects were studied both in high- and low-fat diet conditions in two models, one acute and another chronic model of experimental colitis that resembles gene expression in human inflammatory bowel disease (IBD). In the first experiments in the high fat diet (HFD), we did not observe a beneficial effect of the addition of plant sterols and stanols on the development of acute dextran sulphate sodium (DSS) colitis. In the chronic CD4CD45RB T cell transfer colitis model, we mainly observed an effect of the presence of high fat on the development of colitis. In this HFD condition, the presence of plant sterol or stanol did not result in any additional effect. In the second experiments with low fat, we could clearly observe a beneficial effect of the addition of plant sterols on colitis parameters in the T cell transfer model, but not in the DSS model. This positive effect was related to the gender of the mice and on Treg presence in the colon. This suggests that especially dietary plant sterol esters may improve intestinal inflammation in a T cell dependent manner.

## 1. Introduction

Inflammatory bowel disease (IBD) is a chronic inflammatory condition characterized by recurrent or continuous uncontrolled inflammation of the colon and/or ileum with repetitive relapses of which the etiology is not completely understood. Clinically, there are recurrent attacks of bloody diarrhea, abdominal pain, weight loss, and general malaise. Despite active treatment protocols, in a small percentage of IBD patients, disease progression combined with non-responsiveness to treatment ultimately leads to colorectal cancer or the necessity of surgical intervention. There is ample evidence suggesting that dysfunction of mucosal T cells plays an important role in the pathogenesis of IBD [1], and that the immunosuppressive drugs currently in use, such as 5-ASA drugs, corticosteroids, and azathioprine, inhibit autoreactive T-cell mediated immune responses. However, these drugs are nonspecific, and as such may result in side effects. Also, treatment with more specifically targeted biologicals results in adverse events, including serious infections [2]. Therefore, to improve disease outcome, and especially to enhance the compliance of patients during prolonged treatment regimens, there is a need for novel treatments with fewer side effects.

Plant sterols and plant stanols are natural dietary ingredients, which share structural similarities with cholesterol. The average intake of plant sterols in habitual diets is approximately 250 mg/day, which is mainly derived from vegetable oils, grain products, nuts, seeds, fruits, and vegetables [3]. The intake of plant stanols (the saturated derivatives) originates from the same sources, but is considerably lower [3,4]. Quantitatively, the most abundant plant sterols in the human diet are β-sitosterol, campesterol, and stigmasterol, while plant stanols are less abundant and consist mainly of sitostanol and campestanol [3]. It is well established that plant sterols and stanols interfere with intestinal cholesterol absorption and lower serum low-density lipoprotein (LDL) cholesterol concentrations up to 10% at a daily intake of 2.5 g [3,4].

An intriguing question is whether these dietary ingredients might have effects beyond lowering LDL. In a recent review, we concluded that there is inconsistent evidence for an anti-inflammatory effect of plant sterol and stanol esters in relation to cardio vascular disease (CVD) risk management, whereas effects on immune function in specific disease conditions are likely [5]. In this respect, we have shown that plant sterols and stanols induce a Th1 shift in cultured human peripheral mononuclear blood cells (PBMCs) from asthma patients [6]. The mechanism underlying this effect most likely involves activation of Toll-like receptor 2 (TLR2) and acts via enhancement of IL-2 production and elevation of Treg numbers and function [7]. This observation of elevated Treg numbers and activity prompted us to test the efficacy of plant sterol and stanol consumption in other T-helper skewed conditions, such as IBD. To the best of our knowledge, effects of plant sterols and stanols on intestinal inflammation have thus far not received much attention. There does exist one earlier observation in a small-scale study showing inhibitory effects of free β-sitosterol in TNBS colitis in mice [8]. Moreover, β-sitosterol was dissolved in Tween-80 and orally administered once a day in that study. Under natural conditions however, plant sterols are hardly present as free sterols, and instead almost always as sterol-glycosides, acetylated sterol glucosides, hydroxycinnamate (ferulate) esters, or fatty acid esters [9]. Furthermore, the bioavailability of plant sterols and stanols largely depends on the amount of fat in the diet [10]. We therefore evaluated the effects of plant sterols and stanols in this study, and their fatty acid esters were incorporated into the diet to closely mimic normal physiological conditions. We evaluated the effects both in high- and low-fat dietary conditions in two different models of experimental colitis that were shown to best resemble gene expression in human IBD [11]. We tested this in the chronic T-cell driven T-cell transfer model in which RAG-1−/− mice lacking natural mature B and T-cells are transplanted with CD4^+^CD45RB^hi^ cells, a model resembling Crohn’s disease, as well as in the acute dextran sodium sulfate (DSS) colitis model of epithelial injury.

## 2. Experimental Section

### 2.1. Animals

The Animal Studies Ethics Committee of the University of Amsterdam, The Netherlands, evaluated and approved all experiments (DMO100305 and DMO100126). For the T cell transfer model, male (28.3–34 gram) and female (20.2–25 gram) mice (with a targeted deletion in the gene for RAG1, (RAG-1−/−) on a C57Bl/6 background were used. These mice were bred in the ARIA Amsterdam animal facility in individually ventilated cages. Pathogen-free female C57BL/6n wild type (wt) mice (12 weeks old) obtained from Charles River (Maastricht, The Netherlands) were used as donors for the CD4^+^CD45RB^hi^ and CD4^+^CD45RB^low^ cells (one donor for three transfers). For the DSS experiments pathogen-free female C57BL/6 mice (10 weeks old) obtained from Harlan Sprague-Dawley (Horst, The Netherlands) were used. During the experiments, the mice were randomly housed in individually ventilated cages; maximum 5 mice per cage. We used 10 mice per group based on earlier studies. Here we accepted 25% variation, a power of 80% and an α of 0.05.

### 2.2. Diets

**Figure 1:**
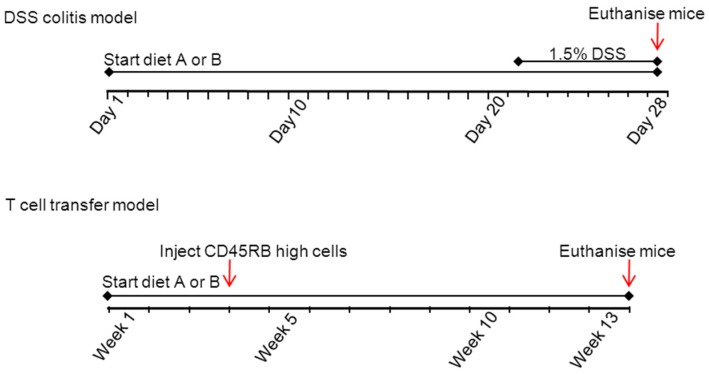
Experimental design of the DSS and T-cell transfer model. The diets are A (based on a HFD) and B (based on a low fat chow diet).

The mice had free access to water and food and were always randomly assigned to groups of 10 mice, each group receiving different diets. In all experiments, the diets (A or B) were given from three weeks prior to the induction of colitis till the moment of sacrificing. For the DSS colitis model, this is on average 21 days diet only followed by 7 days diet plus DSS and in the T-cell transfer model, this is on average 21 days diet only and 10 weeks follow up after injection of CD45RB high cells. In Figure 1 an overview of the experimental set-up is provided.

In the first experiments, mice were randomly allocated to one of the four experimental groups for both experimental models. Three weeks before the start of the induction of colitis, and until the end of the study, the mice received the following diets (diets A, based on a HFD). The first group consumed a plant sterol poor chow (10th percentage of the total energy intake (En%) fat); the second group a plant sterol poor high-fat diet (HFD) based on beef fat containing 41.5 En% fat. The other two groups (three and four) used the same HFD but then enriched with plant sterols (2%) or plant stanols (2%), respectively provided as their rapeseed-oil based fatty acid esters. Small amounts of olive oil, soybean oil, and linseed oil were added to the HFD, but not to the HFD + sterol or stanol esters, to make the amount and type of fatty acids in the three HF diets comparable, since the fatty acids in the sterol and stanol esters (rapeseed oil fatty acids) become available during digestion. The 3.1% plant sterol or stanol esters correspond to ±2% free plant sterols or stanols. The composition of the three experimental high-fat diets is presented in Table 1A.

In the second experiment, the mice were randomly allocated to either the same plant sterol poor chow diet (10 En% fat) as in experiment one, which served as control, or the same plant sterol poor chow diet (diet B) enriched with plant sterol esters (2%) or plant stanol esters (2%). Again, an additional isoenergetic control group consumed the same plant sterol poor diet, containing only the rapeseed oil fatty acids that were normally present as fatty acids in the plant sterol, or stanol fractions that were incorporated as fatty acid esters. The mice received the diets from three weeks before the colitis induction in both models until the end of the study. The composition of the three experimental chow diets, as well as the plant sterol poor diet, is presented in Table 1B. The diets in themselves do not induce loss of body weight, diarrhea or colitis in normal mice.

### 2.3. Experimental Design Mouse Models

DSS colitis was induced by the administration of 1.5% DSS with a molecular weight between 35–55 kD (TdB Consultancy, Uppsala, Sweden) in the drinking water of the mice for seven days. T-cell transfer colitis was induced by intraperitoneal injection of CD4^+^CD45RB^hi^ T cells. These cells were isolated according to a standard protocol [12,13]. In short, single-cell CD4^+^ spleen cells were negatively selected using anti-rat-IgG-coated magnetic beads (Dynal, Life technologies Europe BV, Bleiswijk, The Netherlands) after staining the spleen cells with a depleting antibody mix (anti-mouse B220, CD8 and CD11b). The CD4^+^ T cells were sorted with a FACS ARIA (BD Biosciences, Breda, The Netherlands) after staining with anti-mouse CD45RB FITC and CD4 PerCP (BD Biosciences). CD4^+^CD45RB^hi^ cells were washed and 4 × 10^5^ cells were injected in RAG-1-/- recipient mice. In a control group, 2 × 10^5^ CD4^+^CD45RB^low^ T cells were injected together with the CD4^+^CD45RB^hi^ T cells.

**Table 1:** Composition of the experimental high-fat diets (diet A) and low fat chow diets (diet B).

**A: Composition of the Experimental High-Fat Diets (Diet A)**				
	**HFD ^1^**	**HFD + Plant Sterol Esters**	**HFD + Plant Stanol Esters**	
**Composition (%)**				
Sucrose	39.75	38.97	38.97	
Casein	23.64	23.18	23.18	
Beef fat	15.78	15.47	15.47	
Cellulose	5.91	5.79	5.79	
Olive Oil	2.94	2.07	2.07	
Soybean oil	2.27	2.07	2.07	
Corn Starch	2.59	2.54	2.54	
Vitamin Mix ^2^	0.58	0.58	0.58	
Mineral Mix ^3^	5.44	5.33	5.33	
Choline	0.47	0.46	0.46	
DL Methionine	0.24	0.23	0.23	
Cholesterol ^4^	0.20	0.20	0.20	
Linseed Oil	0.19	-	-	
Plant sterol esters ^5^	-	3.10	-	
Plant stanol esters ^5^	-	-	3.10	
**B: Composition of the Experimental Low Fat Chow Diets (Diet B)**				
	**Chow + Rapeseed Oil ^6^**	**Chow + Plant Sterol Esters**	**Chow + Plant Stanol Esters**	**Chow**
**Composition (%)**				
Sucrose	29.39	29.39	29.39	29.38
Casein	20.00	20.00	20.00	20.00
Cellulose	5.00	5.00	5.00	5.00
Olive Oil	2.83	2.00	2.00	2.00
Soybean oil	2.17	2.00	2.00	2.00
Corn Starch	34.73	32.81	32.81	35.92
Vitamin Mix ^2^	0.58	0.50	0.50	0.50
Mineral Mix ^3^	4.60	4.60	4.60	4.60
Choline	0.40	0.40	0.40	0.40
DL Methionine	0.20	0.20	0.20	0.20
Linseed Oil	0.19	-	-	-
Plant sterol esters ^5^	-	3.10	-	-
Plant stanol esters ^5^	-	-	3.10	-

^1^ HFD: high-fat diet; ^2^ Vitamin mix: vitamins premix, trace elements premix; ^3^ Mineral mix: calcium hydrogen phosphate, calcium carbonate, potassium chloride, potassium dihydrogen phosphate, magnesium sulphate heptahydrate, sodium chloride, magnesium oxide; ^4^ This added amount of 0.2% cholesterol together with the 0.015% cholesterol from beef fat produces a diet that contains 0.22% cholesterol; ^5^ The 3.1% plant sterol or stanol esters correspond to ± 2% free plant sterols or stanols. The chow diet contains ± 10.2 En% fat, whereas the HFD contains ± 41.5 En% fat; ^6^ Rapeseed oil: rapeseed oil fatty acids.

### 2.4. Parameters to Assess Inflammation

The weight of the mice was recorded three times a week for the T cell transfer model and daily in the DSS experiment with the help of an animal caretaker unaware of the composition of the different diets. The humane endpoint for the chronic transfer model was set at a bodyweight loss of more than 15% compared to each animal’s initial weight. The DSS mice were all sacrificed 7 days after the start of the DSS. In both models, spleen and colon were collected after sacrificing. Colons were removed and opened longitudinally. After removing the fecal material, the length and weight of the colons were measured and used as an indicator of disease-related intestinal shortening and thickening, respectively. This was performed with the help of an animal technician unaware of the different dietary treatments.

In the DSS model, the disease activity index (DAI) was assessed by scoring severity of diarrhea, blood in stool, and weight loss at day 7 [14]. Diarrhea was scored on a scale ranging from 0 to 4 as follows: normal stool: 0 points; soft stool: 1 point; very soft stool: 2 points; fluid stool: 3 points; empty, wet colon: 4 points. Blood in stool was scored as follows: no blood: 0 points; blood in stool: 2 points; blood in stool and anal region: 4 points. Weight loss was scored on a scale ranging from 0 to 4 as follows: <1% weight loss: 0 points; 1%–5% weight loss: 1 point; 5%–10% weight loss: 2 points; 10%–15% weight loss: 3 points and >15% weight loss: 4 points. The sum of the scores for diarrhea, blood in stool, and weight loss scores was divided by three to calculate the “average” DAI score, which ranges from 0 (no disease) to 4 (very severe disease). In the CD45RB transfer, we separately scored the diarrhea using a similar 4-point scale as in the DSS model.

### 2.5. Histologic Examination

Longitudinally divided, rolled-up sections of colon were fixed in 4% buffered formalin in PBS for 24 hours and embedded in paraffin for routine histology. Three transverse slices (5 µm) taken from each colonic sample were stained with haematoxylin-eosin and examined by light microscopy. Colonic inflammation was evaluated microscopically in a blinded manner by an experienced pathologist using two different scoring systems. For the DSS model the histology was scored by determining the (1) percentage of involved area; (2) the amount of follicles; (3) oedema; (4) fibrosis; (5) ulcerations; (6) crypt loss; (7) infiltration of granulocytes; and (8) monocytes, all on a 0–3 scale [15].

The percentage of the area that was involved, crypt loss, and ulcerations were scored on a scale ranging from 0 to 3 as follows: 0, normal; 1, less than 10%; 2, 10 to 50%; 3, more than 50%. Follicle aggregates were counted and scored as follows: 0 point, 0–1 follicles; 1 point, 2–3 follicles; 2 point, 4–5 follicles; 3 point, more than 6 follicles. The severity of the other parameters was scored on a scale of 0 to 3 as follows: 0, absent; 1, weak; 2, moderate; 3, severe.

For the T cell transfer model the histology was scored according to the graded histopathological changes scale (current protocols of immunology). Grades were semiquantitatively scored on a scale from 0 (no change) to 4 (most severe). The scoring was based on the mean of 6 criteria: leukocyte infiltration (0: normal, 1: increase in mucosa, 2: increase in mucosa and submucosa, 3: extending into the tunica muscularis); loss of goblet cells (0: none, 1: 10% depletion, 2: 10%–50% depletion, 3: >50% depletion); crypt loss (0: none, 1: 10% loss, 2: 10%–50% loss, 3: >50% loss); epithelial hyperplasia (0: normal, 1: slight, 2: 2–3 times increased crypt length, 3: >3 times increased crypt length); ulceration (0: none, 4: present) and crypt abscesses (0: none, 4: present). As soon as the colon of the mice demonstrated ulceration or abscesses the mice received the maximum score (4).

### 2.6. Immunohistochemical Staining of CD3 and Foxp3

To determine the amount of T cells and Treg cells in the colons of mice that demonstrated an effect after the supplementation, we randomly selected mouse tissue to stain CD3 and Foxp3 using immunohistochemistry. The immunohistological staining of T cells was performed on paraffin-embedded colon tissue with a primary rabbit anti-mouse CD3 (clone SP7, Neomarkers, Thermo Fisher Scientific, Fremont, CA, USA) followed by Power Vision anti-Rabbit HRP (Klinipath, Duiven, The Netherlands) and vector red (Vector Laboratories, Brunswig Chemie, Amsterdam, The Netherlands). The Foxp3 staining was performed on frozen sections with a primary antibody rat anti-mouse Foxp3 (clone FJK-16s, eBioscience, San Diego, CA, USA) followed by rabbit anti-rat biotin (Dako, Glostrup, Denmark) and Dako REAL^TM^ Detection system (K5001, Dako, Glostrup, Denmark). Two independent observers scored the level of T cell infiltration using a semi-quantitative scale from 0–3, where 3 represents extensive infiltration.

### 2.7. Intestinal Tissue Concentrations of Sterols and Stanols

Intestinal plant sterol (sitosterol and campesterol), plant stanol (sitostanol and campestanol), and cholesterol precursor (lathosterol and desmosterol) concentrations were analyzed by gas-liquid chromatography–mass spectrometry (GC-MS), as described previously [16].

### 2.8. Statistical Analysis

All data were analyzed using GraphPad Prism 4 (GraphPad Prism Version 5 for Windows, GraphPad Software, San Diego, CA, USA). Weight curves are expressed as the means ± the standard error of the mean (SEM). All other data were represented in a scatter dot plot with a line representing the mean. Statistical significant (*p* < 0.05) differences between groups was evaluated using different statistical tests. The nonparametric Mann-Whitney test was used for comparing pathology scores, stool scores, Treg scores, CD3 scores and DAI scores. One-way ANOVA with Bonferonni post test was used for comparing colon weights and spleen weights.

## 3. Results

**Figure 2:**
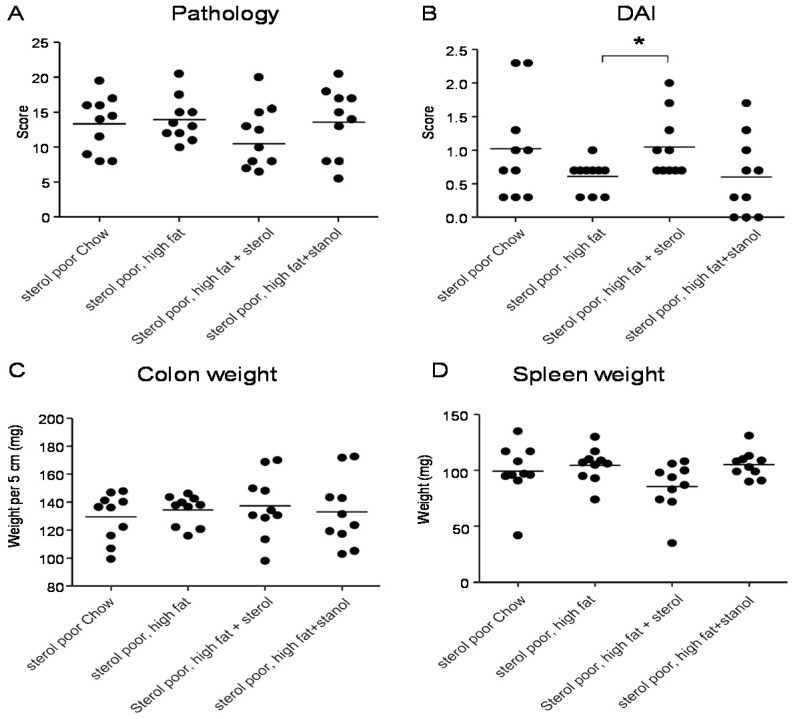
Mice with DSS-induced colitis fed normal chow or a high-fat diet (diet A) supplemented with or without plant sterol or stanol esters. Pathology score (**A**); DAI (**B**); Colon weight (**C**); and Spleen weight (**D**). Each dot represents the data from 1 mouse. * Significant difference between the groups.

**Figure 3:**
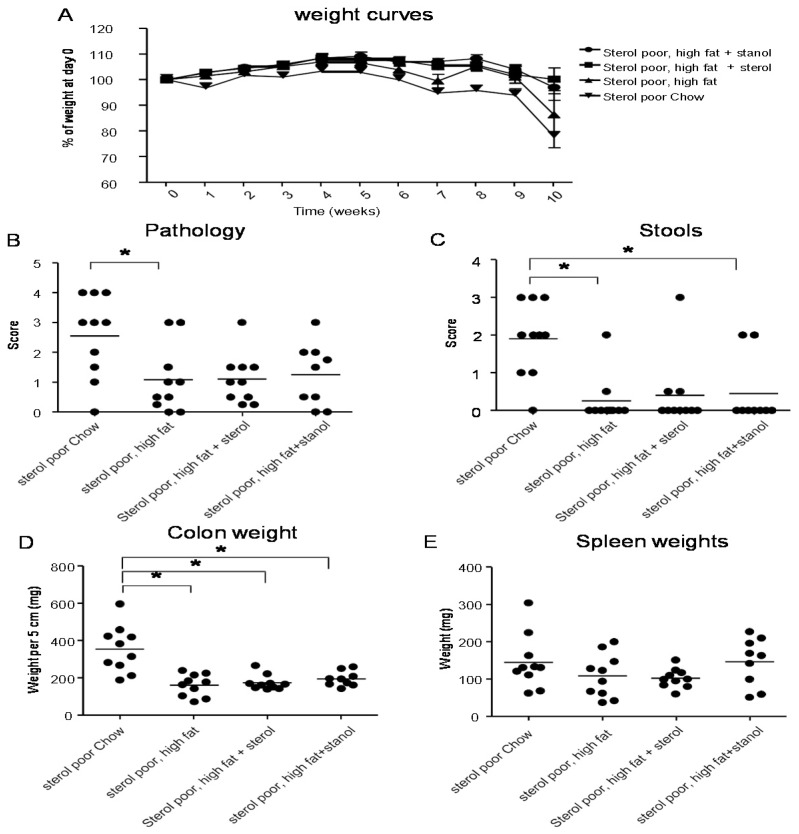
Mice with CD45RB transfer-induced colitis fed normal chow, or a high-fat diet (diet A) supplemented with or without plant sterol or stanol esters. Weight curve (**A**); Pathology score (**B**); Stools (**C**); Colon weight (**D**); and Spleen weight (**E**). Each dot represents the data from 1 mouse. * Significant difference between the groups.

To determine the role of dietary plant sterols and stanols in prevention of intestinal inflammation, we tested for this in two models of experimental colitis, DSS and the CD4^+^CD45RB^hi^ transfer colitis model (T cell transfer model). Our results show that adding plant sterol or stanol esters to the high-fat diets (diet A) did not seem to improve disease severity in the DSS-induced colitis model. In the animals receiving the high-fat diet enriched with added plant sterol, a slight increase in the DAI was observed (Figure 2B), but this did not correspond with an increase in the pathology score and colon weight or a change in spleen weight (Figure 2A,C,D). The disease activity index in the mice that received the additional sterols was enhanced due to a higher percentage of weight loss in this group. The same diets (diet A) in the T cell transfer model demonstrated that a high-fat diet independent of supplementation with plant sterol or stanol esters already gave a significant reduction in the histological score, colon weight, and stools (Figure 3B–D). The addition of plant sterol or stanol esters did not further improve the outcome. The body weight loss and spleen weight did not demonstrate a significant difference between the groups (Figure 3A,E). One mouse in the group supplemented with stanol had to be sacrificed prematurely due to a paralysis. This was not related to the development of colitis and the animal was not included in the analysis.

**Figure 4:**
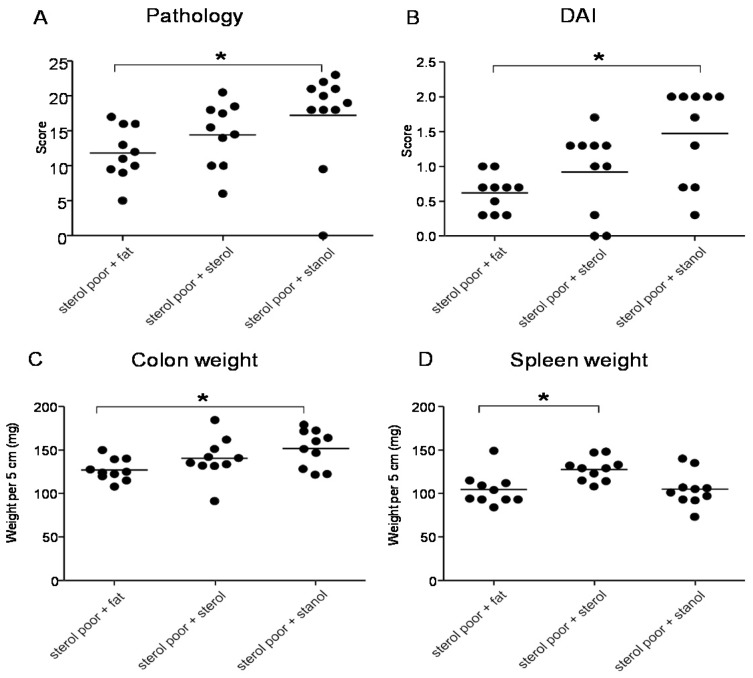
Mice with DSS-induced colitis fed sterol poor chow (diet B) supplemented with or without plant sterol or stanol esters. Pathology score (**A**); DAI (**B**); Colon weight (**C**); and Spleen weight (**D**). Each dot represents the data from 1 mouse. * Significant difference between the groups.

**Figure 5:**
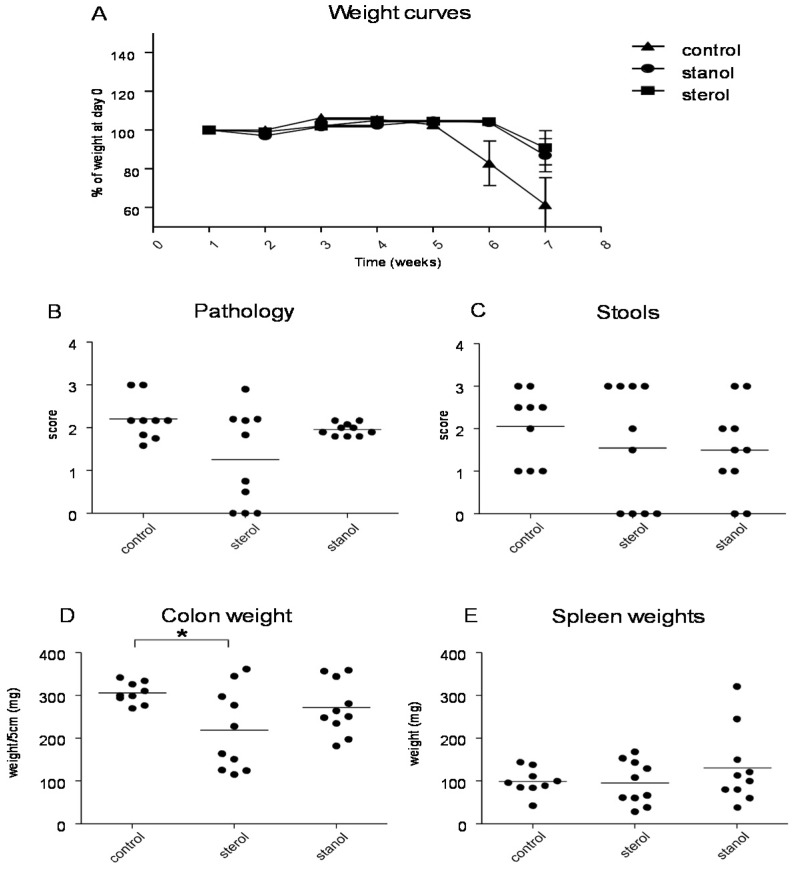
Mice with CD45RB transfer-induced colitis fed sterol poor chow (diet B) supplemented with or without plant sterol or stanol esters. Weight curve (**A**); Pathology score (**B**); Stools (**C**); Colon weight (**D**); and Spleen weight (**E**). Each dot represents the data from 1 mouse. * Significant difference between the groups.

In the next experiments, we tested the effect of plant sterol and stanol on a plant sterol poor chow background (diet B), so without the addition of high fat in the two experimental models of colitis. In both models, a plant sterol poor chow with plant sterol or stanol esters and an iso energetic plant sterol poor chow with added fatty acids were compared. In DSS colitis, there was an increase in the DAI, pathology score, and in colon weight in mice that were fed the diet enriched with plant stanols compared to the control diet (Figure 4A–C). In the mice fed the diet enriched with plant sterols an increased spleen weight was observed (Figure 4D). In the transfer model, we observed that there was less reduction in body weight in the stanol and sterol groups as compared to the control food (Figure 5A). Regarding the other parameters, colon weight was significantly reduced in the plant sterol-fed group (Figure 5D). The pathology score, spleen weight, and stools were not significantly different between the groups, but the latter values demonstrated a large variation (Figure 5B,C,E).

**Figure 6:**
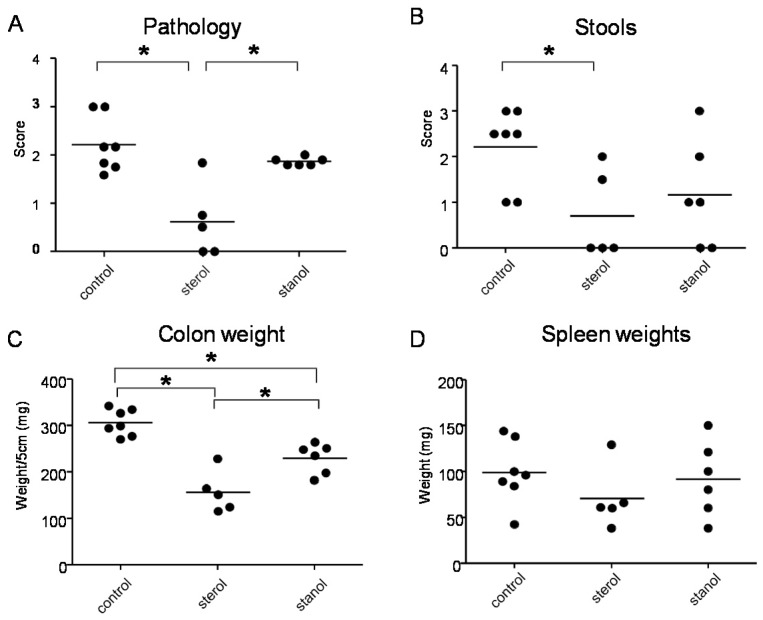
Female mice with CD45RB transfer-induced colitis fed sterol poor chow (diet B) supplemented with or without plant sterol or stanol esters. Pathology score (**A**); Stools (**B**); Colon weight (**C**); and Spleen weight (**D**). Each dot represents the data from 1 mouse. * Significant difference between the groups.

When we separately analyzed the female and male animals, we observed significant differences in the female animals between the different groups. During T cell transfer colitis, animals given both plant sterol or stanol esters were clearly losing less weight as compared to animals receiving high-fat control food. The disease parameters pathology, colon weight and stool were significantly reduced only in the mice on the diet including plant sterol esters (Figure 6A–C), and there was a slight (non-significant) positive effect on spleen weight in this group (Figure 6D).

**Figure 7:**
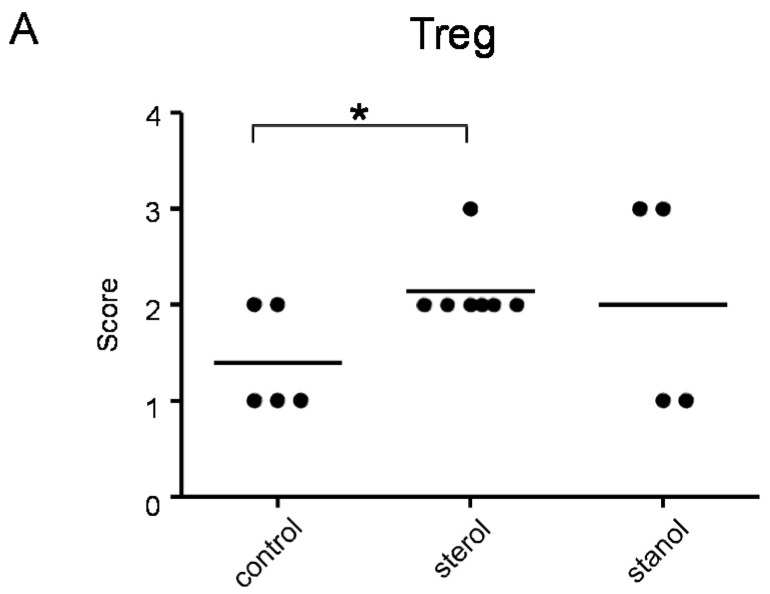

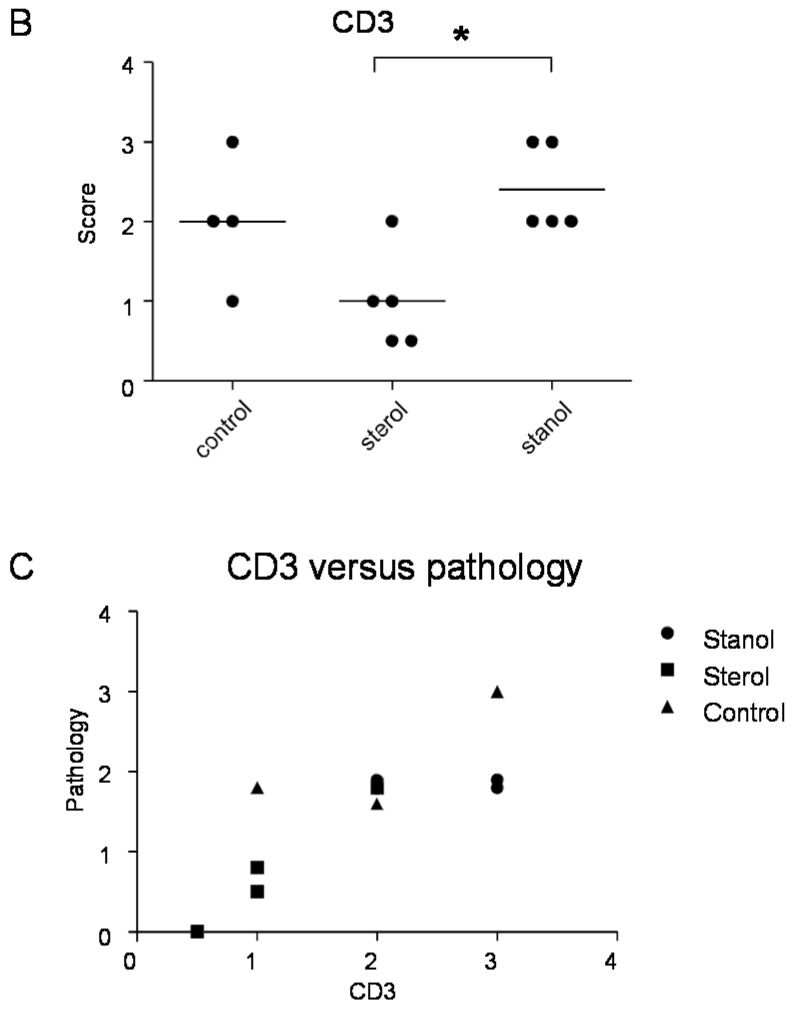
The presence of Treg (**A**) and CD3^+^ T cells (**B**) cells in the colon of mice with CD45RB transfer-induced colitis fed sterol poor chow (diet B) supplemented with or without plant sterol or stanol esters; In (**C**) the correlation between the presence of CD3^+^ T cells and the pathology score is shown. Each dot represents the data from 1 mouse. * Significant difference between the groups.

Since we had previously observed an effect of plant sterol and stanol supplementation on T-cell numbers, although in another disease condition [6], we further determined the number of T cells and Treg cells in colon tissue of mice where we observed a difference in the disease parameters by immunohistochemistry. Infiltrating T cells were seen in all the mice, but differed between the groups. Plant sterol esters reduced the number of CD3-positive cells in the colon compared to the plant stanol ester groups (Figure 7B). The number of CD3-positive T cells correlated with the histological scores (Figure 7C).

**Figure 8:**
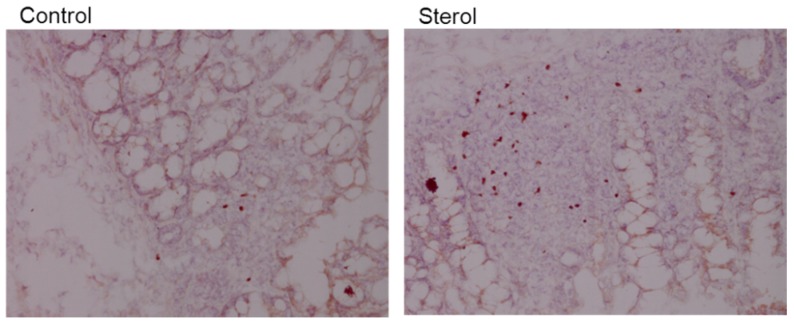
Immunohistochemical staining of Foxp3 in colonic tissue. Left is tissue from a control mouse and right tissue from a mouse fed plant sterol. Magnification 200x.

Plant sterol esters, but not plant stanol esters, significantly increased the number of Treg cells compared to the control diet (Figure 7A and Figure 8). This data suggests that dietary supplementation of plant sterol esters improves T cell dependent intestinal inflammation by decreasing inflammatory CD3 T cells and increasing Treg numbers.

The question becomes whether the effects can be attributed to direct effects of the plant sterols and stanols, or are more likely due to differences in intestinal intracellular cholesterol metabolism. Mice receiving plant sterol-enriched diets showed significantly higher sitosterol and campesterol concentrations in their intestinal samples, and mice receiving stanol ester-enriched diets showed significantly higher sitostanol and campestanol concentrations (Table A1). Interestingly, there was no difference in the intracellular concentrations of cholesterol or the cholesterol precursors lathosterol and desmosterol.

## 4. Discussion

We determined the effect of added dietary plant sterol or stanol esters in a HFD or low-fat diet environment in two different models of experimental colitis (See Table 2 for a summary of the impact of the different diets). In the first experiments with the HFD, we did not observe an effect of the addition of plant sterols and stanols on the prevention of the development of DSS colitis. In the T-cell transfer colitis, we primarily observed a protective effect of the presence of high fat on the development of the colitis. In this HFD condition, however the addition of plant sterol or stanol did not result in any modifying effect. In another model (hemorrhagic shock in rats) however, high-fat enteral nutrition reduced pro-inflammatory responses, resulting in a preserved gut barrier function [17]. This could also play a role in the reduced intestinal inflammation we observed in the transfer model. In the DSS model, DSS administration results in the reduction of the firmly adherent mucosal layer that protects the epithelial layer [18]. This might explain the differences of the HFD-induced reduced inflammation we observe between the two models. There are also discrepancies in the effectiveness of dietary intervention in colitis models using other dietary lipids [19]. Inconsistent findings of the effect of omega-3 fatty acids in different colitis models demonstrate that we need to study the effects of plant sterol or stanol in more depth. Metabolomic analysis could help to get insights in possible molecular processes [19].

In the second set of experiments with low fat, we clearly observed a beneficial effect of the addition of plant sterols on colitis parameters in the T-cell transfer model, but again not in the DSS model. This positive effect was most likely related to an effect on the presence of Tregs in the colon. This suggests that especially dietary plant sterol esters may improve intestinal inflammation in a T cell dependent manner.

**Table 2:** Summary of the impact of the different diets, A and B in the different animals models with and without plant sterol/stanol.

**Diet A**		
Plant sterol/stanol feeding + HFD	DSS colitis	=/↑
	Transfer colitis	↓
HFD alone	DSS colitis	=
	Transfer colitis	↓
**Diet B**		
Plant sterol/stanol feeding + low fat	DSS colitis	↑
	Transfer colitis	↓ (female)
	Transfer colitis Tregs	↑

=: no effect; ↑ : worsened parameters of colitis or increased number of Tregs; ↓ : improved parameters of colitis.

DSS is widely used as an inducer of inflammation in the intestine. It causes damage to the epithelial lining of the intestine which increases the interaction of the microbiota with the intestinal immune system, leading to an acute inflammation mainly involving innate immune cells [20]. The finding that we do not observe a beneficial effect of the administration of plant sterols and stanols in the DSS model might be related to the presence of DSS in the intestine. Although the resulting damage caused by the presence of DSS is rather distal in the colon, we cannot rule out that DSS has also an effect on the rest of the intestine. This might affect bioavailability of the sterols and stanols. Damage to the colon can both decrease or increase absorption of these compounds into the epithelium. In patients with coeliac disease, for example, serum plant sterol concentrations are significantly lower than observed in healthy subjects, most likely attributable to their impaired absorption [21]. Another possibility is that the plant sterols and stanols act via a mechanism that does not underly the DSS-induced pathology.

In the transfer model, we observed a difference between male and female animals in susceptibility to the disease-modifying effect of the addition of sterols, because the effect of plant sterols was more pronounced in female than in male mice. Recently, a similar gender difference was seen in a model of intestinal tumorigenesis [22]. However, the precise mechanisms behind this remain unsolved.

In this study, we can distinguish between the effects of the plant sterol and stanol esters in the two different colitis models, and the effect of the composition of the diets containing high and low fat. Concerning the different models, it has been shown in *in vitro* experiments using cells from asthma patients that plant sterols and stanols influence Treg proliferation and function [6]. In the T cell transfer model, Tregs are important [23]. Our observation that an increase in Tregs after sterol enrichment that is linked to a reduction in disease parameters might indicate that activation of Treg is also relevant in our experiments described here. Another possibility underlying the protective effects of plant sterols and stanols relates to the recent observation by Kim *et al.* [24], suggesting that sitosterol prevents the interaction between lipopolysaccharide (LPS) and toll-like receptor 4 (TLR4) in intestinal macrophages. This would attenuate TLR4 activation and lead to a consequently lower NF-κB activation and a dampened intestinal inflammatory response. If true, this suggests that TLR4 is also important in the phenotypic response in this transfer model. However, we also see the protection of the HFD itself in the transfer model. Given the fact that the HFD was composed mainly of saturated fat (SAFA) from beef fat and it is generally accepted that dietary SAFA’s are able to activate TLR4 [8,25], it is difficult to explain the protection of the HFD, as well as that of sitosterol, via the role of TLR4.

Whether the observed effects of plant sterols and stanols are only “direct effects”, or also partly due to “indirect effects”, is an intriguing question. As we have shown, changes in cellular plant sterol and stanol concentrations in the intestinal wall are visible, suggesting that direct effects could be responsible. The fact that both plant sterols and stanols show protection suggests that both compounds are equally effective in this process. However, this is difficult to explain, since the increase in cellular plant sterol concentrations is much larger when compared to the increase in cellular plant stanol concentrations. In addition, plant stanols lower the concentrations of plant sterols, which implies that plant stanols should be many times more potent than plant sterols at a molar basis, which is possible but not likely. However, the fact that the protective effect of plant sterols appears stronger than the effect of stanols favors a direct effect, in this case, of the plant sterols. The experiment was not designed to confirm this assumption, however, and therefore other explanations deserve further scrutiny. For example, effects could be mediated via changes in cholesterol homeostasis, or changes in microbiota composition. Evidence thus far accumulated shows a clear link between cholesterol homeostasis and adaptive immune responses. In this respect, Glass and Saijo clearly discussed the possibility that targeting cholesterol efflux and synthesis is an interesting target for suppression of antigen driven autoimmune diseases [26]. Moreover, Bensinger and colleagues clearly suggest a role for oxysterol-mediated liver X receptor b (LXRb) activation in T cells, which results in increased cholesterol efflux, and consequently, lower cellular cholesterol levels. In this, the resulting shortage of cholesterol limits membrane biogenesis and, as such, reduces proliferation potential in T-cells [27]. Moreover, Spann *et al.*, recently showed that desmosterol, an intermediate of endogenous cholesterol synthesis, was a key regulator in the inflammatory characteristics of macrophages [28]. Again, there is a prominent role for activation of LXRb (now via desmosterol) resulting in increased cholesterol efflux and dampening of the inflammatory response. In this context, it is likely that plant sterol ester consumption, which is known to influence cellular cholesterol metabolism (lower oxysterol formation plus higher desmosterol formation), might have an effect on this delicate balanced interaction between cholesterol metabolites and the activity of immune cells. In our analysis, however, there were no changes in cellular desmosterol or cholesterol content. Oxysterols were unfortunately not measured due to a lack of material. A second alternative explanation could be found in a difference in intestinal microbiota composition. Indeed an association between the pathogenesis of IBD and gut microbiota composition has been suggested [29]. Patients with colitis showed a dysbiosis as compared to healthy controls. Recently, it was described that plant sterol ester intake induced dramatic shifts in the fecal microbiota composition of hamsters, showing reductions in Coriobacteriacea and Erysipelotrichaceae [30]. Unfortunately, to the best of our knowledge, these effects have never been studied in humans.

## 5. Conclusions

Altogether, these data support the notion that plant sterols and stanols affect several different (patho)-physiological processes. The ultimate challenge is now to see whether these intriguing observations can be extrapolated to the human situation.

## Appendix

**Table A1:** Sitosterol, campesterol, sitostanol, and campestanol concentrations (ng/mg) in intestinal samples.

	Control Group	Sterol Ester Group	Stanol Ester Group
sitosterol (ng/mg)	21.0 ± 13.9	275.2 ± 147.5	15.0 ± 3.5
campesterol (ng/mg)	50.6 ± 23.9	457.5 ± 164.1	40.1 ± 12.1
sitostanol (ng/mg)	7.9 ± 6.0	16.5 ± 9.1	212.3 ± 58.3
campestanol (ng/mg)	5.5 ± 5.6	12.7 ± 6.3	73.2 ± 27.5
